# Cannabinoids and Adverse Convulsive Effects: A Pharmacovigilance and Addictovigilance Analysis of Cases Reported in France

**DOI:** 10.1111/fcp.70028

**Published:** 2025-06-20

**Authors:** Marie‐Laure Laroche, Marion Labetoulle, Emilie Jouanjus, Edeltraut Kröger, Arsène Zongo

**Affiliations:** ^1^ Regional Centre of Pharmacovigilance CHU Limoges Limoges France; ^2^ CEIP‐Addictovigilance CHU de Toulouse Toulouse France; ^3^ Université Toulouse, CERPOP Inserm UMR1295 Toulouse France; ^4^ Population Health and Optimal Health Practices Research Axis CHU de Québec Research Centre—University Laval Research Centre Quebec City Quebec Canada; ^5^ Faculty of Pharmacy University Laval Quebec City Quebec Canada; ^6^ Centre D'excellence sur le Vieillissement de Québec Centre Intégré Universitaire de Soins et Services Sociaux de la Capitale Nationale Quebec City Quebec Canada

**Keywords:** adverse drug reactions, cannabinoids, pharmacovigilance, seizure

## Abstract

**Background:**

Seizures after the use of cannabinoids are reported, but no precise descriptions of the characteristics of subjects and factors that may trigger seizures are available.

**Objectives:**

To study the characteristics and circumstances associated with the occurrence of seizures in individuals using cannabinoids for medical or recreational purposes.

**Methods:**

A retrospective analysis of spontaneous reports of adverse drug effects issued by the French pharmacovigilance and addictovigilance systems, and by manufacturers, extracted data from the Eudravigilance database (01/01/1985–21/07/2023). The request used the broad MedDRA SMQ term ‘convulsive’, with all products containing cannabinoids (THC, CBD, cannabis or natural cannabinoids).

**Results:**

Among 4296 notifications with cannabinoids, 130 (3%) reports of convulsive effects were analysed: 29 cases (23.3%) related to medical use (27 CBD, 1 THC and 2 combined THC/CBD preparations) and 98 (75.4%) related to recreational use. The median age was 29.0 years (min‐max: 3–75), 78.7% were men and 81.1% were serious cases. Among the recreational users, 38.8% used *Cannabis sativa* with a history of epilepsy, and 68.4% of them were taking antiepileptics. In total, 67.7% of individuals had at least one risk factor for seizures, i.e., 31.0% among medical users and 78.6% among recreational users. The main risk factors with medical use were inefficacy of CBD (17.2%), fatigue (13.8%) and concomitant epileptogenic medications (10.3%). The main risk with recreational use was concomitant epileptogenic medications (39.8%), consumption of illicit drugs (33.7%) and alcohol (32.7%).

**Conclusion:**

This analysis demonstrates the importance of alerting cannabinoid users, particularly recreational cannabis users and those with a history of epilepsy, about seizure‐associated risks. Moreover, educational information should be provided together with the prescription of licensed cannabinoids and medical cannabis.

**Abbreviations**
ADR: adverse drug reaction
ANSM: French Medicine Agency
CBD: cannabidiol
EMA: European Medicines Agency
FDA: Food and Drug Administration
GABA: gamma‐aminobutyric acid
HLGT: High Level Group Term
HLT: High Level Term
IQR: interquartile range
LLT: Lower Level Term
MedDRA: Medical Dictionary for Regulatory Activities
PT: preferred term
SMQ: Standardised MedDRA Queries
SOC: System Organ Class
THC: delta‐9 tetrahydrocannabinol

## 1 Introduction

Cannabinoids have been used for medicinal and recreational purposes for a long time in human history, in the form of phytocannabinoids or synthetic derivatives [1]. The effects of cannabinoids are mainly associated with delta‐9 tetrahydrocannabinol (THC), the principal component responsible for the psychoactive effects reported in humans and the cannabidiol (CBD) [2]. The cannabinoid system plays an important role in controlling neuronal excitability and brain function [2].

Initially, the beneficial effects of *Cannabis sativa* on seizures have been observed empirically. Following animal studies and clinical trials, CBD has been approved in the United States in 2018 and in Europe in 2019 for use in patients with severe and medication‐resistant forms of epilepsy (i.e., Dravet syndrome, Lennox–Gastaut syndrome and tuberous sclerosis complex), in association with a first‐line treatment such as clobazam. However, paradoxically, since its approval, undesirable convulsive effects have frequently been reported [3, 4, 5, 6]. Prior to this, seizures had been reported as an adverse effect in clinical trials with CBD in patients with epilepsy [3]. In a systematic review of Fazlollahi et al. in assessing the adverse events of CBD use in patients with epilepsy, the percentage of status epilepticus was higher in the CBD group than in the control group (3.6% vs. 2.7%, but not significantly) [4]. In 2023, Ammendolia et al. conducted a retrospective analysis on the safety of CBD use as an antiepileptic agent using data from the Eudravigilance database, the pharmacovigilance system of the European Medicines Agency (EMA), which allows monitoring the safety of medicines marketed in Europe [5]. In this study, the most common serious adverse reactions reported for the use of CBD were also aggravations of epilepsy (25.5% of reports). Similarly, a study by the same research team showed a frequency of 18.6% for convulsions with the use of CBD, not licensed as a drug for medical use [6]. However, these two pharmacovigilance studies did not analyse the circumstances and risk factors surrounding the adverse effects, thus not allowing for contextualization of the occurrence of seizures.

In addition, rare cases of seizures have been reported with recreational use or accidental ingestion of *C. sativa* by children [7, 8, 9, 10, 11]. In a study from the French Addictovigilance Network, neurological adverse effects with recreational cannabis were the second most frequently reported adverse effect, with 10.7% of them being convulsions [12]. In these reports, the THC content of recreational cannabis was blamed, leading to the suggestion that THC, an agonist of the CB1 receptor (type 1 cannabinoid receptor), might have proconvulsant effects [13]. However, animal models have produced conflicting results on the proconvulsivant and anticonvulsivant effects of THC, depending on the chosen animal model [14, 15].

In clinical practice, physicians observed seizures after the use of medical cannabis and with the recreational use of *C. sativa* . However, there are many biological, environmental and medical factors that favour the occurrence of seizures, such as concomitant alcohol or illicit drug consumption, fever, asthenia, central nervous system infections, traumatic brain injury, stroke, ionic disorders, hypoglycaemia and epileptogenic medications [16]. To date, there are no precise descriptions available of the characteristics of subjects exposed to THC or CBD and experiencing seizures, of the factors that may trigger the seizure, or of the context of the exposure. These are important factors to consider in the management and care of patients with epilepsy, but also for recreational cannabis users. Furthermore, Bueno et al. advocated for a more comprehensive understanding of the safety profile of cannabinoids, emphasising the necessity for real‐world data [17].

The aim of the present study was to investigate personal and product characteristics and circumstances of use that may be related to the occurrence of seizure in subjects using cannabinoids according to the type of use: medical or recreational use, from pharmacovigilance and addictovigilance data.

## 2 Methods

### 2.1 Design

We conducted a systematic pharmacovigilance‐addictovigilance assessment of reports of a seizure with any cannabinoid use, having occurred in France between January 1, 1985 and July 31, 2023. We used the narrative of cases to identify the available individual characteristics of subjects, the cannabinoids and other medications involved, as well as the circumstances of use in relation to the occurrence of seizures.

### 2.2 Overview of Data Sources

#### 2.2.1 Safety Monitoring of Cannabinoids by the French Pharmacovigilance and Addictovigilance Systems

**FIGURE 1:**
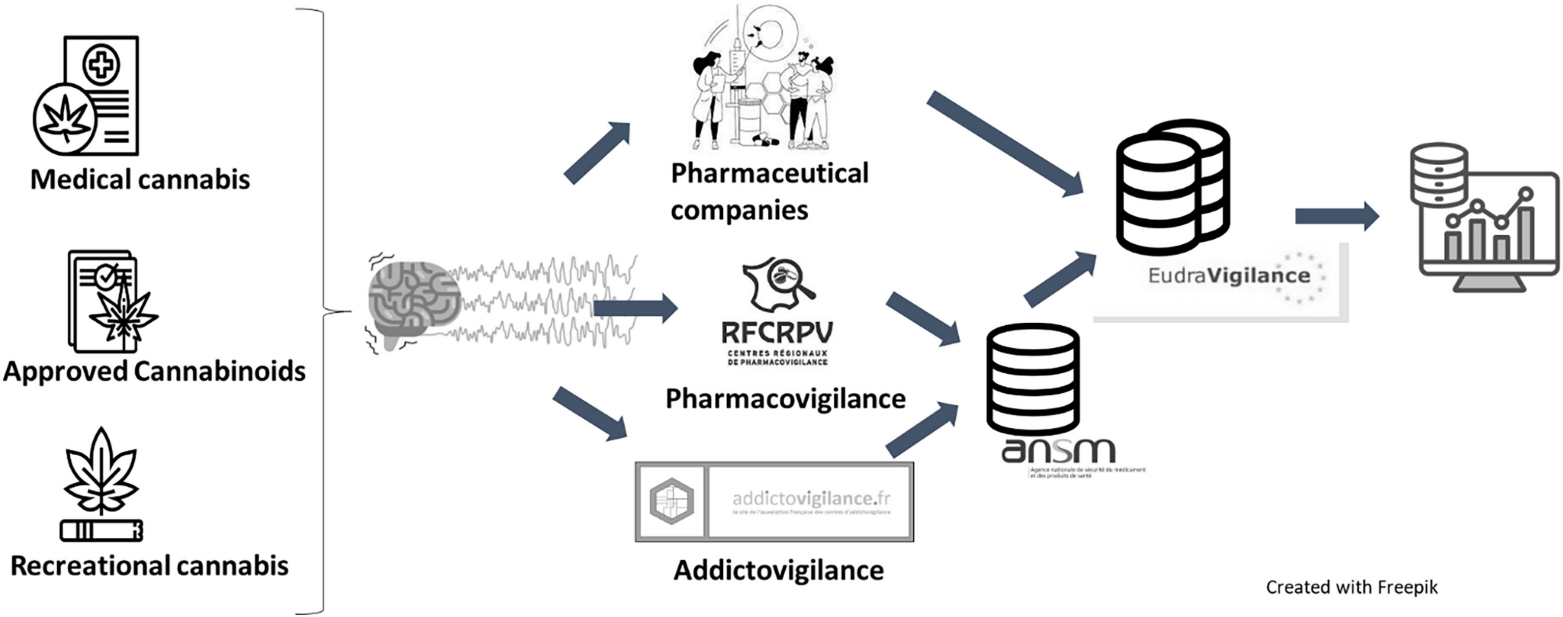
Organisation of the safety monitoring of cannabinoids by the pharmacovigilance and addictovigilance systems in France. *Legend*: Pharmaceutical companies, French Pharmacovigilance centres and French Addictovigilance centres collect, evaluate, record and exploit data on adverse reactions occurring in France. Adverse reactions related to medical cannabis and approved cannabinoids are reported to manufacturers and French Pharmacovigilance centres (RFCPV, reseau français des centres régionaux de Pharmacovigilance). Adverse effects related to cannabis and synthetic cannabinoids, whether used for self‐medication or recreationally, are reported to French Addictovigilance Network. All adverse effects are recorded in the French Pharmacovigilance and Addictovigilance database coordinated by the ANSM, or in the databases of pharmaceutical companies for cases notified solely to them. All cases are then electronically sent to the EudraVigilance database by the ANSM and the pharmaceutical companies, respectively.

In France, cannabinoids are subject to a double control system depending on the type of use: medical or recreational (Figure 1). Cannabinoids for a therapeutic purpose may be used in France under two modalities. On one hand, there are formal licensed pharmaceuticals products that obtained a marketing authorisation or are accessible through early access programmes, e.g., Epidyolex (CBD, cannabidiol) or Marinol (synthetic THC or dronabinol) issued by the French or European health authority. It is estimated that there are around 200 patients/year treated with Epidyolex in France (public data unavailable for Marinol because it is prescribed as a compassionate medication only). On the other hand, medical cannabis, derived from the cannabis plant, is available in the form of flowers for inhalation by vaporisation, of oil or capsules for oral use. These products have different THC/CBD ratios (THC dominant ratio, CBD dominant ratio or balanced THC and CBD ratio) and contents. The French Medicine Agency (ANSM) had assessed a specific experimental programme in order to allow the medical use of cannabis derived from the cannabis plant (2021–2024) [18]. This experimentation was launched for five indications: palliative situations, supportive care in oncology, severe and medication‐resistant forms of epilepsy, painful spasticity associated with multiple sclerosis or other central‐nervous‐system‐related diseases and neuropathic pain [19]. Approximately 3000 patients have been enrolled in the experimental programme, of whom around 8% (250) were suffering from drug‐resistant epilepsy and 50% (1700) from neuropathic pain [18, 19]. On the other hand, the use of cannabis outside the authorised medical framework is illegal in France, with the French regulations, prohibiting cultivation, possession and recreational use, being among the most restrictive in Europe. In Europe, national surveys of cannabis use show that, overall, 8% of European adults (22.8 million, aged between 15 and 64) used cannabis in 2023 [20], and around 1.3% (3.7 million) of adults (aged 15–64) were daily or almost daily cannabis users (i.e., they used the drug for 20 days or more in the previous month). Despite the restrictive regulations on recreational cannabis in France, the prevalence of cannabis use remains among the highest in Europe: In 2021, 47.3% of 18–64‐year‐olds had already experimented with cannabis (32.9% in 2010), with twice as many men as women. While cannabis use remains the most prevalent illicit drug among teenagers, the proportion of adults is also increasing, particularly for daily use [20, 21].

In the context of the adverse effects of cannabinoids, manufacturers, French Pharmacovigilance centres and French Addictovigilance centres are responsible for collecting, evaluating, recording and exploiting data on adverse reactions occurring in France [22, 23]. As an example, the French Addictovigilance Network already explored cannabis‐related cardiovascular disorders [24]. Figure 1 explains the organisation of the safety monitoring of cannabinoids by the pharmacovigilance and addictovigilance system in France.

All cases of adverse reactions are documented, and a causal relationship between events and suspected substances is rigorously evaluated before their recording. For all reports, suspected adverse reactions are evaluated by the pharmacologists of both the Pharmacovigilance and Addictovigilance centres, or the pharmaceutic companies. The assessment of causality in Pharmacovigilance uses a standardised scale of causality assessment developed in France: If a causality can be reasonably suspected between the substance and the adverse drug reaction (ADR), the substance is defined as ‘suspect’ [25]. Adverse reactions are coded using the Medical Dictionary for Regulatory Activities (MedDRA) [26]. The MedDRA dictionary is organised by System Organ Class (SOC), divided into High Level Group Term (HLGT), High Level Term (HLT), Preferred Term (PT) and Lower Level Term (LLT). Cases of adverse reactions are considered as serious if they resulted in death, hospitalisation (or hospitalisation prolongation), permanent incapacity or disability, congenital anomaly/birth defect, if they are life‐threatening or considered as clinically relevant by the physicians reporting the cases.

Regardless of the context of cannabinoid use and how case reports are collected, all adverse effects are finally recorded in the French Pharmacovigilance and Addictovigilance database coordinated by the ANSM, or in the databases of pharmaceutical companies for cases notified solely to them. All cases are then electronically sent to the EudraVigilance database by the ANSM and by the pharmaceutical companies [27] (Figure 1).

### 2.3 Data Extraction

In the aim of collecting all French reports of seizures related to cannabinoids from pharmaceutical companies and French pharmacovigilance‐addictovigilance systems, the Eudravigilance database has been used. The ANSM selected all cases of seizures for which the products CBD, tetrahydrocannabinol, cannabis or natural cannabinoids were coded as ‘suspect’ or ‘interaction’, from January 1, 1985 to July 31, 2023, and reported in France. To identify the adverse reactions, the Standardised MedDRA Queries (SMQ) ‘convulsion’ were used to consider all terms related to a seizure including all forms of epilepsy, convulsion or seizure [28]. The broad SMQ ‘convulsions’ was used to be more sensitive.

The extraction data included the following: identification number of the case, age, sex, name of products, origin of the report (pharmacovigilance, addictovigilance or manufacturer), reporter qualification, year of occurrence, seriousness, outcome of the case and narrative reports. The following data were extracted from the narrative reports: indication, context of use (medical or recreational), history of epilepsy, concomitant antiepileptic treatments and other risk factors for seizure (illicit drugs, alcohol, hyperthermia/fever, central nervous system infection, traumatic brain injury, brain tumour, stroke, hyponatraemia, hypoglycaemia, asthenia, underdosed antiepileptic medication, CBD ineffectiveness and concomitant epileptogenic medications) [16]. In order to identify concomitant epileptogenic medications, we consulted several sources of the literature, such as certain antipsychotics (clozapine, quetiapine, aripiprazole, haloperidol etc.), antidepressants (amitritptyline, citalopram, sertraline, mirtazapine, mianserine etc.), psychostimulants (e.g., gamma‐hydroxybutyrate), opioids (morphine, tramadol etc.) and drugs used in opioid dependence (buprenorphine and methadone), non‐opioid analgesics (diclofenac, indomethacin etc.), antibiotics (penicillin, fluoroquinolones, isoniazid etc.) and chemotherapeutic (cisplatin, oxaliplatin, 5‐fluorouracil, methotrexate etc.) [16, 29, 30, 31].

### 2.4 Statistical Analysis

We calculated the reporting ratio of seizures observed with cannabinoids among all reports on adverse effects of cannabinoids during the study period and described the characteristics of seizures according to the context of use (medical vs. recreational). We also described the frequencies of each risk factor for a seizure identified, according to each context of use. Finally, we calculated the proportion of subjects with a seizure using cannabinoids and presenting at least one risk factor of seizure in each context of use, besides a diagnosis of epilepsy. The only continuous quantitative variable (age) was described by the mean and the corresponding standard deviation (SD), and by the median and the respective interquartile range (IQR). All statistical analyses were performed using Microsoft Excel 2021 (version 16.0).

### 2.5 Ethical Considerations

In accordance with French law (articles 34 and 38 of the law n°78‐17 regarding electronic data use, databases and freedoms) (*loi relative à l'informatique, aux fichiers et aux libertés*), the ANSM guarantees the protection of patients' privacy about the data they collect and the studies they conduct using these data for their pharmacovigilance missions. Thus, no specific ethical approval had to be obtained for this study.

## 3 Results

A total of 4332 case reports involving CBD and/or THC products were recorded in France between January 1985 and July 2023, including 143 cases of seizures related to CBD and/or THC products were extracted. Thirteen cases were excluded from the analysis due to coding errors or a lack of narrative. Of the 130 complete cases, three were accidental intoxications with *C. sativa* in children aged 1 to 2 years. All three children remained drowsy and hypotonic after their epileptic seizure. They were hospitalised to monitor their neurological condition, which improved. In total, we thus described 127 cases of convulsions after the exclusion of cases related to the accidental use of cannabinoids during the study period, which represented a proportion of 3.0% of seizures observed with cannabinoids among all reports on adverse effects of cannabinoids. This proportion ranged between 2% and 7% according to the years of reporting (Appendix A).

Seizures after the cannabinoid use were mainly reported by healthcare professionals (physicians, pharmacists or others) (*n* = 123, 96.9%) and more frequently in the context of recreational use or abuse (*n* = 98, 77.2%) (Table 1), as compared to a total of 29 (22.8%) cases of seizures reported in the context of medical use, mainly with CBD. Globally, seizures were considered severe in 81.1% of cases, with hospitalisations more common in recreational than in medical use (57.1% vs. 20.7%). Eight deaths were observed, equally divided between medical and recreational use. The four deaths associated with licenced cannabinoids occurred in patients with severe epilepsy: Two were sudden unexplained death in epilepsy (SUDEP) in patients with Lennox–Gastaut syndrome (aged 7 and 33 years); one in a 3‐year‐old child with Dravet syndrome who died of status epilepticus associated with a disturbed hepatic and haemostasis profile and who was being treated with combined CBD/THC purchased over the Internet despite medical advice to the contrary (toxicological analysis of the product with no identified toxins). Another death occurred in a 3‐year‐old child with encephalopathy associated with a mutation in the CACNA1E gene, who had been treated with CBD and clobazam for 3 years, with a recent introduction of levetiracetam and lamotrigine 6 months earlier, who died of an epileptic seizure with cardiopulmonary arrest. The four other deaths associated with recreational CBD occurred in users of several illicit drugs (polydrug users) who died after a tonic–clonic seizure with cardiorespiratory arrest or multivisceral failure (aged 34–41 years).

**TABLE 1:** Characteristics of reports with cannabinoid‐related seizures (*n* = 127).

	Medical use (*n* = 29)	Recreational use (*n* = 98)	Total (*n* = 127)
Origin of reports *n* (%)			
Addictovigilance	0 (0.0%)	92 (93.8%)	92 (72.4%)
Pharmacovigilance	13 (44.8%)	3 (3.1%)	16 (12.6%)
Manufacturer	16 (55.2%)	3 (3.1%)	19 (15.0%)
Reporter qualification *n* (%)			
Physician	21 (72.4%)	64 (65.3%)	85 (67.0%)
Pharmacist	3 (10.3%)	28 (28.6%)	31 (24.4)
Consumer	2 (7.0%)	2 (2.0%)	4 (3.1%)
Others health professionals	3 (10.3%)	4 (4.1%)	7 (5.5%)
Seriousness *n* (%)			
Serious reports	22 (75.9%)	81 (82.6%)	103 (81.1%)
Hospitalisation	6 (20.7%)	56 (57.1%)	62 (48.8%)
Death	4 (13.8%)	4 (4.1%)	8 (6.3%)
Life threatening	2 (6.9%)	8 (8.1%)	10 (7.9%)
Other medically important condition	10 (34.5%)	13 (13.3%)	23 (18.1%)
Type of products *n* (%)			
*Cannabis sativa* (recreational)	0 (0.0%)	35 (36,1%)	35 (27.6%)
*C. sativa* (abuse)	0 (0.0%)	62 (63,9%)	62 (48.8%)
Cannabidiol (CBD)	26 (89.7%)	1 (1.0%)	27 (21.2%)
THC	1 (3.4%)	0 (0.0%)	1 (0.8%)
Combined THC/CBD preparations	2 (6.9%)	0 (0.0%)	2 (1.6%)

Individuals with a seizure after the cannabinoid use were predominantly male, especially for recreational use (Table 2). Overall, the median age was 29.0 years (min‐max: 3–75), but there were more children in the reports related to the medical use than in the reports on recreational use (53.0% and 4.1% were under 16 years of age, respectively). The main medical indication was severe epilepsies for medical use (Table 2). In the recreational users, 38.8% used *C. sativa* with a history of epilepsy, and 68.4% of these users were also taking antiepileptic medications. The main antiepileptic medications used among those using cannabinoids for medical purposes were valproate (*n* = 11), lamotrigine (*n* = 10) and clobazam (*n* = 9) (Table 3). Among the case reports concerning recreational use, the main antiepileptic medications were valproate (*n* = 11), followed by levetiracetam (*n* = 7) and lamotrigine (*n* = 6) (Table 3).

**TABLE 2:** Characteristics of individuals with cannabinoid‐related seizures (*n* = 127).

	Medical use (*n* = 29)	Recreational use (*n* = 98)	Total (*n* = 127)
**Age (years)**			
Mean ± SD	23.4 ± 17.2	32.0 ± 12.3	30.1 ± 13.9
Median (IQR)	21.5 (11.0–33.0)	30.0 (22.5–39.0)	29.0 (20.0–38.0)
Min‐max	3–75	11–75	3–75
Sex *n* (%)			
Female	10 (34.5%)	17 (17.3%)	27 (21.3%)
Male	19 (65.5%)	81 (83.7%)	100 (78.7%)
Medical indication of cannabinoid use *n* (%)			
Lennox–Gastaut syndrome	10 (34.4%)		
Dravet syndrome	3 (10.4%)		
Drug‐resistant epilepsy	5 (17.2%)		
Other type of epilepsy	4 (13.8%)		
Epileptic encephalopathy	3 (10.4%)		
Bourneville tuberous sclerosis	1 (3.4%)		
Neuropathic pain	3 (10.4%)		
History of epilepsy *n* (%)			
Yes	27 (93.1%)	38 (38.8%)	
No	2 (6.9%)	46 (46.9%)	
Unknown	0 (0.0%)	14 (14.3%)	
Concomitant antiepileptic medications	21 (72.4%)^a^	26 (68.4%)^b^	
Number of antiepileptic medications			
1	4 (19.0%)	20 (76.9%)	
2	4 (19.0%)	3 (11.5%)	
3	9 (42.9%)	2 (7.7%)	
4	3 (14.3%)	1 (3.9%)	
5	1 (4.8%)	0 (0.0%)	
At least one risk factor of seizure	9 (31.0%)	77 (78.6%)	86 (67.7%)
Consumption of illicit drugs^c^	0 (0.0%)	33 (33.7%)	33 (26.0%)
Alcohol	0 (0.0%)	32 (32.7%)	32 (25.2%)
Hyperthermia, fever	2 (6.9%)	4 (4.6%)	6 (4.7%)
Central nervous system infection	0 (0.0%)	1 (1%)	1 (0.8%)
Traumatic brain injury	0 (0.0%)	2 (2%)	2 (1.6%)
Brain tumor	0 (0.0%)	2 (2%)	2 (1.6%)
Stroke	0 (0.0%)	3 (3%)	3 (2.4%)
Hypoglycemia	0 (0.0%)	11 (11.2%)	11 (8.7%)
Hyponatremia	1 (3.4%)	3 (3.1%)	4 (3.1%)
Fatigue	4 (13.8%)	19 (19.4%)	23 (18.1%)
Inefficacy of CBD	5 (17.2%)	0 (0.0%)	5 (3.9%)
Underdosing of antiepileptic medication	1 (3.4%)	9 (9.2%)	10 (7.9%)
Concomitant epileptogenic medication	3 (10.3%)	39 (39.8%)	42 (33.1%)

^a^ Antiepileptic medications were used by 19 patients with severe epilepsy and 2 patients with neuropathic pain.

^b^ Antiepileptic medications among recreational users of cannabinoids with a history of epilepsy.

^c^ Illicit drugs were used by 33 individuals and polysubstance abuse was common (cocaine [19], ecstasy [5], heroin [41], LSD (diethyllysergamide) [38], GHB (gama‐hydroxybutyric acid) [1], 3MMC (mephedrone) [1], and methamphetamine [1]).

**TABLE 3:** Number of concomitant antiepileptic medications and epileptogenic medications in individuals with cannabinoid‐related seizures.

	Medical use	Recreational use
Number of concomitant antiepileptic medications (*n* = 92)	56 (60.9%)	36 (30.1%)
Calcium ion channel modulators	0 (0.0%)	2 (5.5%)
Pregabaline	0	2
Sodium ion channel modulators	21 (37.5%)	15 (41.7%)
Carbamazepine	3	2
Eslicarbazepine	1	1
Lacosamide	1	4
Lamotrigine	10	6
Oxcarbazepine	1	1
Phenytoin	0	1
Rufinamide	1	0
Zonisamide	4	0
Enhancers of GABAergic transmission	15 (26.8%)	1 (2.8%)
Clobazam	9	1
Clonazepam	4	0
Phenobarbital	1	0
Stiripentol	1	0
Modulators of presynaptic machinery (SV2A)	4 (7.1%)	7 (19.4%)
Levetiracetam	4	7
Selective postsynaptic inhibitors of excitatory neurotransmission (AMPA receptor)	1 (1.8%)	0 (0.0%)
Perampanel	1	0
Multiple modes of action	14 (25.0%)	11 (30.5%)
Sodium ion channel, GABA_A_ receptors, NMDA receptors: felbamate	1	0
Sodium ion channel, AMPA/kainite receptors, GABA_A_ receptors: topiramate	2	0
Sodium ion channel, GABA turnover, NMDA receptors: valproate	11	11
Number of concomitant epileptogenic medications (*n* = 53)	3 (5.7%)	50 (94.3%)
Opioids	1 (33.3%)	14 (28.0%)
Morphine	1	3
Tramadol	0	11
Antipsychotics	0 (0.0%)	8 (16.0%)
Aripiprazole	0	1
Cyamemazine	0	3
Haloperidol	0	1
Quetiapine	0	1
Risperidone	0	2
Antidepressants	2 (66.7%)	9 (18.0%)
Nonselective monoamine reuptake inhibitors		
Amitriptyline	0	2
Amoxapine	0	1
Selective serotonin reuptake inhibitors		
Escitalopram	1	0
Paroxetine	0	1
Sertraline	0	2
Others antidepressants		
Mianserine	0	1
Minalcipran	1	0
Mirtazapine	0	2
Psychostimulants	0 (0.0%)	1 (2.0%)
Gamma‐hydroxybutyrate (GHB)	0	1
Drug used in opioid dependence	0 (0.0%)	16 (32.0%)
Buprenorphine	0	10
Methadone	0	6
Antihistaminics	0 (0.0%)	1 (2.0%)
Desloratadine	0	1
Antinematodal agents (veterinary drug)	0 (0.0%)	1 (2.0%)
Levamisole^a^	0	1

Abbreviations: AMP, alpha‐amino‐3èhydroxy‐5‐methyl‐6‐isoxaloepropionic acid; GABA gamma‐aminobutyric acid; NMDA, N‐methyl‐D‐aspartate; SV2A, synaptic vesicle glycoprotein 2A.

^a^ Levamisole combined with cocaine reduces the seizure threshold [54].

Among the whole sample of case reports, two‐thirds (67.7%) of individuals with a seizure after the cannabinoid use had at least one seizure risk factor besides epilepsy. The proportions were 31.0% among those with medical use and 78.6% among those with recreational use (Table 2). The main risk factors among medical use cases were inefficacy of CBD (17.2%), fatigue (13.8%) and concomitant epileptogenic medications (10.3%), including mainly morphine and antidepressant drugs. The main risk factors among those with recreational use were concomitant epileptogenic medications (39.8%) including mainly buprenorphine and methadone, opioids, antidepressants and antipsychotics, followed by consumption of illicit drugs (33.7%, the first illicit drug was cocaine) and alcohol (32.7%) (Table 2).

## 4 Discussion

This pharmacovigilance assessment of seizures occurring in subjects using cannabinoids showed that cases are observed both in a context of medical and recreational use, and that their reporting was rare among cases of cannabinoid‐related adverse reactions (3%). Among the whole sample of 127 case reports of seizures as adverse effects in subjects using cannabinoids, two‐thirds (67.7%) of individuals had at least one concomitant risk factor of seizures: 31.0% among those with medical use and 78.6% among those with recreational use.

Our results showed that a majority of reported cases of seizures were among those using cannabis recreationally (77%), as compared to medical use (23%). The number of patients treated with licenced cannabinoids is low in France (200 patients/year with Epidyolex, 3200 patients in the experimental context since 2021), explaining the low number of cases reported in the context of medical use in France. More cases of seizures related to recreational cannabis use were reported in relation to the high prevalence of cannabis use in France, which has among the highest number of cannabis users in Europe [20]. Moreover, in our study, the characteristics of cannabis‐related seizures were similar to those of the general population of recreational cannabis users, in terms of sex (81% in our study compared with 82% in the French population of cannabis users) and age (mean age of 32.0 in our study compared to 54% subjects under 35 years in the population of cannabis users in France) [20]. In a survey examining the reasons for self‐medicated use associated with self‐reported cannabis use, a majority of people aged 50 and over reported medical versus recreational use of cannabis (52% vs. 18%, respectively), while the younger 18–29 age group reported a majority of recreational versus medical use (18% and 50%, respectively) [32]. In our sample, 53% of seizure cases among medical users were children, compared to 4.1% among recreational users.

In contrast to studies conducted in other pharmacovigilance systems, our study showed a lower proportion of seizures after the cannabinoid use among all cannabinoid‐related reported adverse events cases in France (3%). A pharmacovigilance study of adverse reactions to Epydiolex recorded in the FDA's FAERS (Adverse Event Reporting System) database between 2018 and 2023 showed that one third of reported ADRs were related to seizures [33]. In Europe, including French data, similar results were observed in the Eudravigilance database, where seizures accounted for 25.5% of reports with licensed cannabinoid and 19% with recreational cannabis [5, 6]. First of all, a majority of seizure reports occurred among persons with a history of epilepsy (51.2%). The low proportion of seizure cases among all cannabinoid‐related reports of adverse events, specifically in France, may be explained by a different reporter profile compared to other countries. Healthcare professionals are the main reporters in the French pharmacovigilance‐addictovigilance system [23, 34]. They may generally consider seizures as a symptom of the lack of treatment efficacy and thus not consider and report them as adverse reactions. In pharmacovigilance, the lack of therapeutic efficacy is part of the definition of ADRs. Thus, information on this suspicion should be collected and reported. Given the ambiguity for healthcare professionals of considering a seizure among patients treated for epilepsy as either a lack of treatment efficacy or as an adverse event, seizure events may be largely under‐reported, due to a lack of comprehensive knowledge of the pharmacovigilance definitions. Complicating healthcare professionals understanding of pharmacovigilance definitions is the fact that therapeutic ineffectiveness refers to the notion of efficacy (benefit) for a healthcare professional, and not to adverse reactions (risk). Another reason for the low number of reports may be that patients treated with cannabis use for medical purposes are under medical supervision, which may reduce the risk of adverse effects. We observed a relatively high number of reports in the addictovigilance data. A seizure is not perceived as the result of a cannabinoid dependence effect and is likely to be reported more frequently to the addictovigilance system.

Several factors may explain why cannabinoids and particularly CBD may be associated with seizure events. One of those factors may be related to the origins of seizures. Shorvon et al. proposed an etiologic classification of epilepsies in four categories: idiopathic, symptomatic, provoked and cryptogenic [35]. In our analysed reports, symptomatic and provoked categories may explain most of the cases. Indeed, symptomatic epilepsy is defined as an epilepsy of an acquired or genetic cause, associated with gross anatomic or pathologic abnormalities and/or clinical features, indicative of underlying disease or condition. In this category, seizures can be due to acquired causes such as brain infection, trauma, stroke or tumour, encountered in 9% of cases in our data. Similarly, a provoked seizure is defined as an epilepsy in which a specific systemic or environmental factor is the predominant cause of the seizures, and in which there are no gross causative neuroanatomic or neuropathologic changes. Most cases of provoked seizure are caused by fever, menstrual cycle, sleep–wake cycle, metabolic and endocrine; 51% of cases in our data were related to these factors. Cannabinoids, by their excitatory activity on the brain, could thus be the cause of provoked seizures. Other provoked seizures are considered as reflex epilepsies, induced by precipitating factors such as visual, auditive or somatosensory stimuli. In our study, most individuals had associated factors that could have triggered the onset of seizures. These factors differed according to the context of cannabinoid use. However, co‐use of epileptogenic medications was the most common factor in both contexts of use. Psychotropic drugs and morphine were common in both types of use, and opiate substitution drugs (methadone and buprenorphine) in recreational use. Medication‐related factors include blood–brain barrier penetration, target pharmacological activity on brain receptors/proteins, dose level, route of administration and lipid solubility [16]. Mechanisms of medication‐induced seizures could be impacts to metabolic pathways (e.g., ibuprofen, diclofenac and indomethacin), cell death due to neurotoxicity (e.g., cisplatin, methotrexate, interferon alpha, busulfan and 5‐fluorouracil) or altered function/expression of voltage gated ion channels or neurotransmitter interferences (e.g., penicillin G, carbapenems, cephalosporins, fluoroquinolones, clozapine, opioids and lidocaine).

Among recreational cannabis users in our sample, the use of other illicit drugs and alcohol was the second most common risk factors for seizures, after the use of epileptogenic medications. These findings are not surprising, given that individuals who use cannabis recreationally are also more likely to use other illicit substances and alcohol [12, 36, 37]. Illicit drugs associated with cannabinoid use, such as cocaine, amphetamines, ecstasy and LSD, are known to cause epileptic seizures. These users may not be sufficiently informed about the risks of seizures associated with illicit drugs and multiple drug use. In addition, THC itself is thought to be a proconvulsant, although this has not been clearly explained [10]. If this effect of THC is confirmed, it could also contribute to explaining why in our data more recreational cannabis‐related cases of seizure are reported than licensed cannabinoids and medical cannabis‐related cases.

Epilepsy is a neurological disorder characterised by an imbalance between the inhibitory neurotransmitter gamma‐aminobutyric acid (GABA) and the excitatory neurotransmitter glutamate. Cannabinoids can modulate GABA transmission, since endocannabinoid binding at presynaptic type 1 cannabinoid (CB1) receptors results in the suppression of GABA‐mediated inhibition from GABAergic neurons [38, 39]. CBD is thought to act as an antagonist or even a negative allosteric modulator of the CB1 receptor, explaining its use as anticonvulsant agent [40]. However, other findings suggest an absence of direct interaction of CBD at this receptor and rather point to an indirect action mediated by different other mechanisms [41]. The observation of seizure as adverse effect related to the medical use of cannabinoids, particularly CBD, indicated for treatment‐resistant epilepsy forms, is counterintuitive and hard to explain. Previous research showed that some patients with epilepsy treated with CBD may have an increase in the number of seizures, which is a paradoxical effect [42, 43] and is currently unexplained. It is suggested that the use of non‐pure CBD products (i.e., potentially containing other cannabinoids) may be linked to an increase in seizures compared to the use of the CBD product approved by the Food and Drug Administration (FDA), which does not contain other cannabinoids [44]. For example, in a study that assessed the effect of CBD‐enriched products (i.e., containing some amount of THC) for intractable paediatric epilepsy [44], a seizure aggravation was reported in 7% of the patients [44]. However, while contamination may contribute to explaining some cases related to some preparation types of CBD, it cannot explain cases related to Epydiolex, which contains purified CBD. Other hypotheses such as the interaction of CBD with other epileptogenic medications or factors should be explored for such cases. Further hypotheses suggest that the effect of CBD could depend on whether it is combined with other antiseizure medications or not. In a preclinical study that assessed the interaction between CBD and other antiseizure medications in a rat model of recurrent severe seizures, the combination of CBD with the antiseizure medications reduced the severity and prevalence of generalised seizures. In contrast, CBD alone reduces the seizure severity, but does not decrease the expression of generalised seizures [45]. Due to the magnitude of CBD's pharmacokinetic interaction, the combination of this drug with other antiseizure medications has been suggested to be associated with a proseizure effect in certain contexts [46]. CBD may also attenuate the anticonvulsant effect of levetiracetam due to drug–drug interaction [47]. However, the majority of preclinical studies suggest a potentiation of the antiseizure medication effect while combined with CBD [47, 48, 49, 50]. Indeed, the complex pharmacology of CBD includes direct and indirect interactions at CB1 receptors, and both CBD antagonism and potentiation of THC have been suggested [41].

The individuals in the study were also exposed to anti‐epileptic drugs, which may also lead to worsening of seizures, with an increase of the frequency or severity of existing seizures, emergence of new types of seizure or the occurrence of status epilepticus. Discrimination of drug‐induced worsening of seizures from spontaneous fluctuation or epilepsy evolution is not always easy. For example, epilepsies may be severely aggravated by inappropriate treatment due to an adverse interaction between the mode of action of the drug and the pathogenetic mechanisms underlying specific seizure types or syndromes. For example, seizure aggravation has been reported in Lennox–Gastaut syndrome with gabapentin, carbamazepine and intravenous clonazepam [51, 52]. Finally, other circumstances such as drug–drug interactions, under‐dosing and lack of treatment adherence may explain the recurrence of seizures.

The strengths of this pharmacovigilance assessment lie in the availability and the analyses of the narrative reports of cases. These narrative reports provide a more accurate clinical description of the circumstances of the occurrence of seizures after the cannabinoid use. The French pharmacovigilance system is one of the largest ADR reporters in the world, and the addictovigilance system is unique in Europe [23, 34]. Moreover, as in France, most reporters are healthcare professionals, the reported cases of seizures have a high degree of accuracy (i.e., validated seizure diagnosis).

Limitations of the study include the spontaneous nature of the reporting in the pharmacovigilance and addictovigilance system leading to possible under‐reporting. A likely important contributing factor to the under‐reporting of cannabis‐related seizures in these French data is their interpretation as resulting from a lack of efficacy rather than as a proper ADR. The level of investigation into the causes of seizures and the quality of the data collected may exclude some risk factors associated with cannabinoid‐related seizures. In particular, co‐exposure medication data, therapeutic indication or context of medication use are likely lacking, or heterogeneously recorded in these adverse reaction reporting databases. Nonetheless, even if the used data are incomplete, they are reported by healthcare professionals and investigated by pharmacologists in the pharmacovigilance and addictovigilance centres before being recorded into the database. Moreover, paradoxical adverse reactions, such as seizures after seizure treatment with CBD, have been shown to be difficult to detect in pharmacovigilance databases [53]. However, even if this possible under reporting of paradoxical adverse effects might be the case for CBD prescribed to treat epilepsy (i.e., medical use of CBD), there is no reason to expect such an underreporting bias for seizure cases with recreational cannabis. Finally, it is not possible to conclude if cannabinoids are the cause of seizures, as other risk factors of seizure were also present among the studied cases. The objective of the study was not to assess a causal relationship between seizure and cannabinoids, but to characterize the profile of cannabinoid users who have seizures, so that preventive measures can be taken.

## 5 Conclusion

The analysis of pharmacovigilance and addictovigilance data provides relevant real‐life information on seizures occurring after the cannabinoid use and their associated risk factors. This highlights the importance of informing cannabinoid users, particularly recreational cannabis users or those with a history of epilepsy, about the associated risks of seizures. The study also highlights factors that are likely to increase the risk of seizures, underlining the importance of healthcare providers carefully assessing the presence of these risk factors before prescribing cannabis products to patients with epilepsy or at high risk of seizure. The findings suggest that individuals at high risk of seizures who use cannabis recreationally or for those with a history of epilepsy should avoid this use without a medical assessment. This is particularly important given the strong pressure to liberalise the use of cannabis in countries where it is still illegal for recreational use and the increase of self‐medication with cannabis. In addition, the prescription of licensed cannabinoids and medical cannabis should be accompanied by educational information to prevent or minimise these adverse effects.

## Author Contributions

**Marie‐Laure Laroche:** conceptualization, methodology, formal analysis, data curation, writing – original draft, writing – review and editing. **Marion Labetoulle:** formal analysis, data curation, writing – review and editing. **Emilie Jouanjus:** writing – review and editing. **Edeltraut Kröger:** writing – review and editing. **Arsène Zongo:** writing – review and editing.

## Disclosure

The figure has been designed using royalty‐free images from the Freepix website and with the permission of the structures concerned by a logo (ANSM, EMA, RFCRPV, Addictovigilance).

## Ethics Statement

In accordance with French regulations, formal approval by an investigational review board is not required for this type of study. No additional approval was required for studies based on the Eudravigilance database because all records respect the patient's and notifier's anonymity. Thus, this observational study did not require patient consent or ethics committee approval.

## Consent

As all data recorded in the Eudravigilance database are anonymous, informed consent is waived.

## Conflicts of Interest

The authors declare no conflicts of interest.

## Data Availability

The approval to access the anonymised data maintained by the French Network of Pharmacovigilance and Addictovigilance requires the data to be treated as confidential with protected and secure access. For this reason, the data cannot be shared publicly.

## Proportions of reports of seizures related to cannabinoids among all reports of adverse effects with cannabinoids by year, France, 1985–2023

**Table:**

	Number of case reports with cannabinoids	Number of seizures related to cannabinoids	Proportions of seizures with cannabinoids among all reports with cannabinoids
1985–2014	45	1	2%
2015	14	1	7%
2016	49	2	4%
2017	107	4	4%
2018	194	4	2%
2019	304	11	4%
2020	428	13	3%
2021	1042	37	4%
2022	1370	39	3%
Until 07/31/2023	779	18	2%

## References

[1] S. Pisanti and M. Bifulco , “Modern History of Medical Cannabis: From Widespread use to Prohibitionism and Back,” Trends in Pharmacological Sciences 38, no. 3 (2017 Mar): 195–198. 28095988 10.1016/j.tips.2016.12.002

[2] M. R. Amin and D. W. Ali , “Pharmacology of Medical Cannabis,” Advances in Experimental Medicine and Biology 1162 (2019): 151–165. 31332738 10.1007/978-3-030-21737-2_8

[3] O. Devinsky , J. H. Cross , L. Laux , et al., “Trial of Cannabidiol for Drug‐Resistant Seizures in the Dravet Syndrome,” New England Journal of Medicine 376, no. 21 (2017): 2011–2020. 28538134 10.1056/NEJMoa1611618

[4] A. Fazlollahi , M. Zahmatyar , M. ZareDini , et al., “Adverse Events of Cannabidiol Use in Patients With Epilepsy: A Systematic Review and Meta‐Analysis,” JAMA Network Open 6, no. 4 (2023): e239126. 37079302 10.1001/jamanetworkopen.2023.9126 PMC10119734

[5] I. Ammendolia , C. Mannucci , L. Cardia , et al., “Pharmacovigilance on Cannabidiol as an Antiepileptic Agent,” Frontiers in Pharmacology 14 (2023): 1091978. 36843933 10.3389/fphar.2023.1091978 PMC9950105

[6] F. Calapai , E. Esposito , I. Ammendolia , et al., “Pharmacovigilance of Unlicensed Cannabidiol in European Countries,” Phytotherapy Research 38, no. 1 (2024): 74–81, 10.1002/ptr.8028. 37800192

[7] S. Zaheer , D. Kumar , M. T. Khan , P. R. Giyanwani , and F. Kiran , “Epilepsy and Cannabis: A Literature Review,” Cureus. 10, no. 9 (2018 Sep): e3278. 30443449 10.7759/cureus.3278 PMC6235654

[8] K. Detyniecki and L. Hirsch , “Marijuana Use in Epilepsy: The Myth and the Reality,” Current Neurology and Neuroscience Reports 15, no. 10 (2015 Oct): 65. 26299273 10.1007/s11910-015-0586-5

[9] M. Spadari , M. Glaizal , L. Tichadou , et al., “Accidental Cannabis Poisoning in Children: Experience of the Marseille Poison Center,” Presse Medicale (Paris, France: 1983) 38, no. 11 (2009 Nov): 1563–1567. 19541448 10.1016/j.lpm.2009.03.020

[10] J. L. Bonkowsky , D. Sarco , and S. L. Pomeroy , “Ataxia and Shaking in a 2‐Year‐Old Girl: Acute Marijuana Intoxication Presenting as Seizure,” Pediatric Emergency Care 21, no. 8 (2005 Aug): 527–528. 16096599 10.1097/01.pec.0000173349.38024.33

[11] J. A. Tilelli and L. D. Spack , “Marijuana Intoxication Presenting as Seizure—Comment,” Pediatric Emergency Care 22, no. 2 (2006): 141, 10.1097/01.pec.0000204831.96139.52. 16481936

[12] E. Bouquet , S. Pain , C. Eiden , et al., “Adverse Events of Recreational Cannabis Use Reported to the French Addictovigilance Network (2012‐2017),” British Journal of Clinical Pharmacology 87, no. 10 (2021 Oct): 3925–3937. 34282851 10.1111/bcp.14812

[13] E. E. Kaczor , K. Greene , J. Zacharia , L. Tormoehlen , M. Neavyn , and S. Carreiro , “The Potential Proconvulsant Effects of Cannabis: A Scoping Review,” Journal of Medical Toxicology 18, no. 3 (2022): 223–234, 10.1007/s13181-022-00886-3. 35352276 PMC9198115

[14] S. W. Stadnicki , U. Schaeppi , H. Rosenkrantz , and M. C. Braude , “Delta9‐Tetrahydrocannabinol: Subcortical Spike Bursts and Motor Manifestations in a Fischer Rat Treated Orally for 109 Days,” Life Sciences 14, no. 3 (1974): 463–472. 4823985 10.1016/0024-3205(74)90361-0

[15] P. Martin and P. Consroe , “Cannabinoid Induced Behavioral Convulsions in Rabbits,” Science 194, no. 4268 (1976): 965–967. 982057 10.1126/science.982057

[16] E. A. Larson , M. V. Accardi , Y. Zhong , D. Paquette , and S. Authier , “Drug‐Induced Seizures: Considerations for Underlying Molecular Mechanisms,” International Journal of Toxicology 40, no. 5 (2021 Oct): 403–412. 34514888 10.1177/10915818211040483

[17] L. Bueno , A. Batalla , M. Balcells , and H. López‐Pelayo , “Ensuring Safety in Cannabinoid Prescriptions: A Call for Critical Assessment,” European Neuropsychopharmacology: The Journal of the European College of Neuropsychopharmacology 87 (2024): 27. 39032391 10.1016/j.euroneuro.2024.06.006

[18] ANSM , “Mise en Place de L'expérimentation du Cannabis Médical [Internet],” (2024), [cited 2024 Jul 5]. https://ansm.sante.fr/dossiers‐thematiques/cannabis‐a‐usage‐medical/mise‐en‐place‐de‐lexperimentation‐du‐cannabis‐medical.

[19] ANSM , “Cannabis à Usage Médical [Internet].” (2024), [cited 2024 Jul 5]. https://ansm.sante.fr/dossiers‐thematiques/cannabis‐a‐usage‐medical.

[20] EMCDDA , “Cannabis—The Current Situation in Europe (European Drug Report 2024) The Interne.” 2024, https://www.euda.europa.eu/publications/european‐drug‐report/2024/cannabis_en.

[21] S. F. Rezag Bara , M. Mary‐Krause , S. Wallez , and J. S. Cadwallader , “Experience of Cannabis Use From Adolescence to Adulthood in France: An Interpretative Phenomenological Analysis,” International Journal of Environmental Research and Public Health 20, no. 5 (2023): 4462. 36901478 10.3390/ijerph20054462 PMC10002113

[22] E. Jouanjus , J. Micallef , M. Mallaret , and M. Lapeyre‐Mestre , “Comment on: An Insight Into Z‐Drug Abuse and Dependence: An Examination of Reports to the European Medicines Agency Database of Suspected Adverse Drug Reactions,” International Journal of Neuropsychopharmacology 22, no. 8 (2019): 528–530. 31194866 10.1093/ijnp/pyz033 PMC6672683

[23] J. Micallef , É. Jouanjus , M. Mallaret , and M. Lapeyre Mestre , “Détection des Signaux du Réseau Français D'addictovigilance : Méthodes Innovantes D'investigation, Illustrations et Utilité Pour la Santé Publique,” [Safety Signal Detection by the French Addictovigilance Network: Innovative Methods of Investigation, Examples and Usefulness for Public Health] Thérapie 74, no. 6 (2019): 579–590, 10.1016/j.therap.2019.09.005. 31694770

[24] E. Jouanjus , M. Lapeyre‐Mestre , and J. Micallef , “Cannabis Use: Signal of Increasing Risk of Serious Cardiovascular Disorders,” Journal of the American Heart Association 3, no. 2 (2014): e000638. 24760961 10.1161/JAHA.113.000638 PMC4187498

[25] Y. Arimone , I. Bidault , J. P. Dutertre , et al., “Réactualisation de la Méthode Française D'imputabilité des Effets Indésirables des Médicaments,” Thérapie 66, no. 6 (2011): 517–525, 10.2515/therapie/2011073. 27393472

[26] E. G. Brown , L. Wood , and S. Wood , “The Medical Dictionary for Regulatory Activities (MedDRA),” Drug Safety 20, no. 2 (1999 Feb): 109–117. 10082069 10.2165/00002018-199920020-00002

[27] “EudraVigilance—European Database of Suspected Adverse [Internet],” [cited 2024 Jul 12]. https://www.adrreports.eu/index.html.

[28] “2.19 Convulsions (SMQ). Introductory Guide for Standardised MedDRA Queries (SMQs) Version 26.0,” (2024).

[29] A. W. Hitchings , “Drugs That Lower the Seizure Threshold,” Adverse Drug Reaction Bulletin 298, no. 1 (2016): 1151–1154, 10.1097/FAD.0000000000000016.

[30] E. Kumlien and P. O. Lundberg , “Seizure Risk Associated With Neuroactive Drugs: Data From the WHO Adverse Drug Reactions Database,” Seizure 19, no. 2 (2010 Mar): 69–73. 20036167 10.1016/j.seizure.2009.11.005

[31] Micromedex [Internet], “Merative,” (2024), http://www.micromedexsolutions.com.

[32] M. Rotermann and M. M. Pagé , “Prevalence and Correlates of Non‐Medical Only Compared to Self‐Defined Medical and Non‐Medical Cannabis Use, Canada, 2015,” Health Reports 29, no. 7 (2018): 3–13. 30020531

[33] Q. Zhou , Z. Du , K. Qu , et al., “Adverse Events of Epidiolex: A Real‐World Drug Safety Surveillance Study Based on the FDA Adverse Event Reporting System (FAERS) Database,” Asian Journal of Psychiatry 90 (2023): 103828. 37949044 10.1016/j.ajp.2023.103828

[34] L. Aagaard , J. Strandell , L. Melskens , P. S. G. Petersen , and E. Holme Hansen , “Global Patterns of Adverse Drug Reactions Over a Decade: Analyses of Spontaneous Reports to VigiBase,” Drug Safety 35, no. 12 (2012): 1171–1182. 23072620 10.1007/BF03262002

[35] S. D. Shorvon , “The Etiologic Classification of Epilepsy,” Epilepsia 52, no. 6 (2011): 1052–1057. 21449936 10.1111/j.1528-1167.2011.03041.x

[36] J. P. Connor , M. J. Gullo , G. Chan , R. M. Young , W. D. Hall , and G. F. X. Feeney , “Polysubstance Use in Cannabis Users Referred for Treatment: Drug Use Profiles, Psychiatric Comorbidity and Cannabis‐Related Beliefs,” Frontiers in Psychiatry 4 (2013): 79, 10.3389/fpsyt.2013.00079. 23966956 PMC3736050

[37] G. W. Smith , M. Farrell , B. P. Bunting , J. E. Houston , and M. Shevlin , “Patterns of Polydrug Use in Great Britain: Findings From a National Household Population Survey,” Drug and Alcohol Dependence 113, no. 2–3 (2011): 222–228. 20863629 10.1016/j.drugalcdep.2010.08.010

[38] A. Ludányi , L. Eross , S. Czirják , et al., “Downregulation of the CB1 Cannabinoid Receptor and Related Molecular Elements of the Endocannabinoid System in Epileptic Human Hippocampus,” Journal of Neuroscience: The Official Journal of the Society for Neuroscience 28, no. 12 (2008): 2976–2990. 18354002 10.1523/JNEUROSCI.4465-07.2008 PMC6670708

[39] K. Z. Peters , J. F. Cheer , and R. Tonini , “Modulating the Neuromodulators: Dopamine, Serotonin, and the Endocannabinoid System,” Trends in Neurosciences 44, no. 6 (2021 Jun): 464–477. 33674134 10.1016/j.tins.2021.02.001 PMC8159866

[40] E. C. Rosenberg , P. H. Patra , and B. J. Whalley , “Therapeutic Effects of Cannabinoids in Animal Models of Seizures, Epilepsy, Epileptogenesis, and Epilepsy‐Related Neuroprotection,” Epilepsy & Behavior 70, no. Pt B (2017): 319–327, 10.1016/j.yebeh.2016.11.006. 28190698 PMC5651410

[41] J. M. McPartland , M. Duncan , V. Di Marzo , and R. G. Pertwee , “Are Cannabidiol and Δ(9)‐Tetrahydrocannabivarin Negative Modulators of the Endocannabinoid System? A Systematic Review,” British Journal of Pharmacology 172, no. 3 (2015): 737–753, 10.1111/bph.12944. 25257544 PMC4301686

[42] S. A. Hussain , R. Zhou , C. Jacobson , et al., “Perceived Efficacy of Cannabidiol‐Enriched Cannabis Extracts for Treatment of Pediatric Epilepsy: A Potential Role for Infantile Spasms and Lennox‐Gastaut Syndrome,” Epilepsy & Behavior 47 (2015 Jun): 138–141. 25935511 10.1016/j.yebeh.2015.04.009

[43] C. A. Press , K. G. Knupp , and K. E. Chapman , “Parental Reporting of Response to Oral Cannabis Extracts for Treatment of Refractory Epilepsy,” Epilepsy & Behavior 45 (2015): 49–52, 10.1016/j.yebeh.2015.02.043. 25845492

[44] M. Tzadok , S. Uliel‐Siboni , I. Linder , et al., “CBD‐Enriched Medical Cannabis for Intractable Pediatric Epilepsy: The Current Israeli Experience,” Seizure 35 (2016 Feb): 41–44. 26800377 10.1016/j.seizure.2016.01.004

[45] C. L. Frías‐Soria , D. Pérez‐Pérez , S. Orozco‐Suárez , and L. Rocha , “Cannabidiol Modifies the Seizure Expression and Effects of Antiseizure Drugs in a Rat Model of Recurrent Severe Seizures,” Seizure 90 (2021 Aug): 67–73. 33879386 10.1016/j.seizure.2021.04.008

[46] C. Zavala‐Tecuapetla , H. Luna‐Munguia , M. L. López‐Meraz , and M. Cuellar‐Herrera , “Advances and Challenges of Cannabidiol as an Anti‐Seizure Strategy: Preclinical Evidence,” International Journal of Molecular Sciences 23, no. 24 (2022): 16181. 36555823 10.3390/ijms232416181 PMC9783044

[47] K. Socała , E. Wyska , M. Szafarz , D. Nieoczym , and P. Wlaź , “Acute Effect of Cannabidiol on the Activity of Various Novel Antiepileptic Drugs in the Maximal Electroshock‐ and 6 Hz‐Induced Seizures in Mice: Pharmacodynamic and Pharmacokinetic Studies,” Neuropharmacology 158 (2019): 107733. 31377197 10.1016/j.neuropharm.2019.107733

[48] S. H. Chuang , R. E. Westenbroek , N. Stella , and W. A. Catterall , “Combined Antiseizure Efficacy of Cannabidiol and Clonazepam in a Conditional Mouse Model of Dravet Syndrome,” Journal of Experimental Neurology 2, no. 2 (2021): 81–85, 10.33696/neurol.2.040. 34308420 PMC8301289

[49] G. Cabral‐Pereira , D. Sánchez‐Benito , S. M. Díaz‐Rodríguez , et al., “Behavioral and Molecular Effects Induced by Cannabidiol and Valproate Administration in the GASH/Sal Model of Acute Audiogenic Seizures,” Frontiers in Behavioral Neuroscience 14 (2020): 612624, 10.3389/fnbeh.2020.612624. 33551767 PMC7862126

[50] E. R. Kundrick , B. M. Marrero‐Rosado , M. de Araujo Furtado , M. Stone , C. R. Schultz , and L. A. Lumley , “Cannabidiol Reduces Soman‐Induced Lethality and Seizure Severity in Female Plasma Carboxylesterase Knockout Mice Treated With Midazolam,” Neurotoxicology 82 (2021): 130–136, 10.1016/j.neuro.2020.12.002. 33290784 PMC7856212

[51] N. A. Gayatri and J. H. Livingston , “Aggravation of Epilepsy by Anti‐Epileptic Drugs,” Developmental Medicine and Child Neurology 48, no. 5 (2006 May): 394–398. 16608550 10.1017/S0012162206000843

[52] P. Genton , “When Antiepileptic Drugs Aggravate Epilepsy,” Brain & Development 22, no. 2 (2000 Mar): 75–80. 10722956 10.1016/s0387-7604(99)00113-8

[53] Y. Hakimi , N. Petitpain , V. Pinzani , J. L. Montastruc , and H. Bagheri , “Paradoxical Adverse Drug Reactions: Descriptive Analysis of French Reports,” European Journal of Clinical Pharmacology 76, no. 8 (2020 Aug): 1169–1174. 32418024 10.1007/s00228-020-02892-2

